# Extending a Hand: Corruption and Solidarity with the Less Privileged Domestically and Beyond

**DOI:** 10.1007/s12116-022-09352-1

**Published:** 2022-05-13

**Authors:** Fredrik G. Malmberg

**Affiliations:** Faculty of Social Sciences, Business and Economics, Åbo Akademi University, Turku, Finland

**Keywords:** Corruption perceptions, Domestic solidarity, Global solidarity, Moderation

## Abstract

Social cohesion, often operationalized using measures of generalized social trust, has received enormous amounts of attention in previous scholarly work. However, another dimension of this broad phenomenon, norms of social solidarity, has meanwhile largely been overlooked in previous research. This study analyzes the association between micro-perceptions of corruption and solidarity with the less privileged both domestically and beyond, and how this association might vary across different societies with different types and forms of corruption. The data come from ISSP Citizenship II and include 33 countries, analyzed with multilevel regression models. The results show that the link between individual corruption perceptions and global solidarity varies so that it is comparatively weak and positive in contexts judged as more corrupt according to the Corruption Perception Index, while it is strong and negative in contexts judged by experts as relatively corruption free. For domestic solidarity, in turn, there is some evidence of a comparatively weak positive association but no significant contextual variations.

## Introduction

A famous quotation often attributed to Mahatma Gandhi reads, “A civilization is measured by how it treats its weakest members.” This well-known and often quoted phrase sets the stage for the topic of this article, which is the relationship between perceptions of corruption and solidarity with those who are worse off, both domestically and beyond on a global level.

The question concerning the association between corruption and social solidarity has received abundant attention in recent years in academic circles. Many studies have argued that corruption causes societal fragmentation, erodes social solidarity, and makes ordinary citizens less willing to trust and co-operate with people outside their own in-group (Bigoni et al. 2016; Rothstein and Uslaner 2005; Rothstein and Eek 2009; Rothstein 2013; Rothstein 2017; Svallfors, 2013), thereby making it more difficult to solve so-called collective action problems (Ostrom, 1998). These problems are presumably likely to be further exacerbated on the inter- and supranational levels, where globalism and growing cross-border interdependence have accentuated the weaknesses and limitations of nation-states in tackling problems like climate change and viral pandemics. UN Secretary-General António Guterres, for instance, recently called for “global solidarity to ensure that every person, everywhere, has access [to the COVID-19 vaccine]” (UN News 2020).

While many cross-national empirical studies have rigorously analyzed the determinants of social trust, which is viewed by some as a vital source of social solidarity (Rothstein and Uslaner 2005; Rothstein, 2017), surprisingly few have focused on what is argued to be additional dimensions of “social cohesion” (Ariely 2014), such as norms of social solidarity. Even fewer have differentiated between social solidarity on a domestic level, i.e., the willingness to help fellow compatriots who are worse off, and solidarity on a global level, i.e., the willingness to help non-compatriots who are worse off than you are. The ambition of this article is to test the theory that perceptions of corruption erode social solidarity by focusing directly on norms of social solidarity.

This article proposes that the influence of individual corruption perceptions on both domestic and global solidarity is contingent on the overall quality of governmental institutions. More precisely, it argues that the negative association between corruption perceptions and the willingness to help less privileged people is considerably stronger in societies with well-performing institutions, where perceptions of corruption are more likely to express anti-establishment sentiments pitting the “corrupt elite” against the “pure people,” rather than actual experiences of corruption. Furthermore, it also argues that the strength of these associations is dependent on the *type* of solidarity (domestic or global), where the negative effect is especially strong for solidarity with less privileged people in other countries, at least partly due to the antiglobalist and nativist sentiments and dissatisfaction with prevailing domestic conditions inherent to these (often radical right) populist views.

This study examines these questions with multilevel regression using data from the International Social Survey Programme (ISSP) Citizenship II and including 33 countries (ISSP, 2016). The article proceeds as follows. First, I discuss the central concepts of the study to develop hypotheses on the associations between corruption perceptions and the norm importance of asymmetrical social solidarity. In the following section, I present the data and variables before turning to the empirical analyses themselves. Finally, I discuss the conclusions drawn from these results.

The results suggest that there are indeed considerable differences depending on the type of solidarity in question. While there is a robust and relatively strong negative association between perceptions of corruption and global solidarity, the results for domestic solidarity are considerably weaker and non-significant in most cases. Furthermore, the former association can primarily be found in contexts where conventional corruption is commonly viewed as quite rare and institutional quality conversely seen as quite high.

## Corruption and Solidarity with the Less Privileged, Domestically and Beyond

Corruption, defined by Johnston (2005) as *the abuse of public roles or resources for private benefit*, is a multifaceted phenomenon often associated with acts such as bribe taking and embezzlement of public funds. However, some scholars argue that corruption does not vary just quantitatively, but also qualitatively, i.e., that corruption is not the same thing everywhere (see e.g., Johnston, 2005). Hence, one could also argue that the meaning of *perceptions* of corruption, and the sentiments related to them, can vary across different societies (Bauhr and Charron, 2020). Van de Walle (2008), for instance, has posited that perceptions of corruption in contexts characterized by more ambiguous forms of corruption invisible to ordinary citizens, i.e., less outright bribe taking and embezzlement (petty corruption), most likely have their roots in feelings of political alienation and broader sentiments of mistrust toward elites and elite institutions (and their “pet projects” such as the EU, free trade agreements, or foreign aid).

The word “solidarity,” in turn, is said to originate from Roman Law and the juridical term *obligation in solidum*, meaning that a group is collectively liable for all individual group member debts (Tranow 2019: 26). Solidarity among citizens is generally held to be a desirable feature, not least because of its instrumental value in helping to solve collective action problems, protecting vulnerable groups and constraining inequality (Miller, 2017). Individuals and groups demonstrating a high solidarity might be more willing to accept policies, such as higher taxation or redistribution of funds among regions within the EU, that may be costly for themselves and their home countries but may bring benefits to the community as a whole (Bayram, 2017; Bauhr and Charron, 2020; VanHeuvelen, 2017). Like corruption, solidarity is a complex multi-layered concept with several different meanings and types (see e.g., Althammer, 2019; Tranow, 2019), and hence, it is of essence to clarify what the term refers to in this article.

Previous studies have used varying methods of operationalizing solidarity and social cohesion. Some have utilized survey items concerning social or generalized trust as proxies for solidarity (Rothstein, 2017; Rothstein and Uslaner, 2005; Rothstein and Eek, 2009) while others have used items measuring the respondents’ support for welfare and redistribution policies (Bauhr and Charron, 2020; Peyton, 2020; Svallfors, 2013; VanHeuvelen, 2017). Others in turn concentrate on the aggregate level, looking at the association between various system-level indicators such as the Corruption Perceptions Index (CPI) or the International Country Risk Guide (ICRG) and the welfare regime (Dahlström et al. 2013; Rothstein et al. 2012). Here, I use a more direct strategy revolving around norm importance.

The object of interest in this study concerns solidarity norms, which can be viewed as a subset of civic norms, which in turn can be understood in line with Almond and Verba (1963) and Dalton (2008: 78) as “a shared set of expectations about the citizen’s role in politics” or norms of good citizenship (Denters et al. 2007; Zmerli, 2010). Solidarity norms here refer to a person’s willingness to help persons who are worse off or less privileged. In other words, the focus of this study lies on the micro-level or, in the words of Tranow (2019: 29), “a personal *commitment* toward solidarity norms” (italics in original). Here, I have opted for Tranow’s (2019: 31) definition of solidarity norms as “*expressed expectations that actors ought to provide a transfer of personally controlled resources without compensation in favor of other individuals or a community in certain situations* (italics in original; see also Hechter, 1988).

Of the four solidarity norms^1^ identified by Tranow (2019) as instrumental for solving so-called “critical transfer situations,” the object of interest in this article corresponds closest to *support norms*. Support norms require an actor to provide “sufficient” support to other individuals or groups in need even if there are not enough personal incentives to do so.^2^ Moreover, one could argue that this particular situation demands what he calls a “strong solidarity sacrifice”^3^ due to the asymmetry in the relationship between the aid giver and the aid receiver(s). There is an inequality in resources suggesting that the aid giver cannot expect the aid receiver to reciprocate the aid in case the former ever finds himself in a similar need of aid (Tranow 2019: 50–51). However, he might instead trust society *in general* and/or public institutions to provide support and thereby feel more inclined to “repay” society by offering his own support to others who are worse off. Alternatively, he may feel compelled by religious or ideological norms to act in an altruistic way.

Could perceptions of corruption be expected to affect people’s commitment toward solidarity norms and if so, how? Solidarity is often interpreted as a “manifestation of trust” (Miller, 2017: 67; see also Putnam 2000) since social trust is claimed to be “a key facilitator of collective actions, social solidarity, and social cohesion” (Habibov et al. 2017: 32). While the exact causal relationship between trust and solidarity is difficult to disentangle, one could still reasonably claim that the theoretical distance between these two concepts is relatively short since people who show concern for the welfare of other people are also likely to trust them to reciprocate, or at least not abuse, this trust (Miller, 2017). Empirical support for this argument is provided by studies demonstrating a positive association between generalized social trust and the importance of solidarity norms (Kotzian, 2014; Zmerli, 2010). Hence, the same theoretical arguments and mechanisms used to explain the effects of corruption (perceptions) on social trust could potentially be extended to explain the relationship between the former and solidarity norms.

According to institutional theory, people’s perceptions of their fellow citizens (or people in general) are strongly influenced by the performance of public institutions and the incentive structures created by them (Rothstein and Stolle, 2008; Rothstein and Eek, 2009). This is argued to apply, especially to the institutions tasked with implementing public policies (e.g., the police and the civil service) in an impartial and efficient manner following liberal democratic norms. People who receive fair and impartial treatment from public bureaucrats and officials are claimed to generalize from that experience and conclude that other people also tend to be trustworthy and helpful. In contrast, if they perceive public services as endemically corrupt and feel forced to take part in the corrupt practices themselves, they are likely to draw conclusions from both their own, their fellow citizens’ and public servants’ behavior that other people are not generally to be trusted (Rothstein and Uslaner, 2005). Hence, they should become less likely to feel solidarity with others outside their own in-group. They might assume that few would voluntarily choose to help them in their hour of need, unless it is in their own immediate self-interest, and thusly become less willing to provide help, *ceteris paribus*.

Following the logic of the institutional theory of solidarity outlined above, perceptions of clean, well-functioning public institutions could also be presumed to function as a source of social solidarity in general while perceptions of corruption could in turn act to weaken norms of solidarity. Based on these considerations, the following two hypotheses are formed:

1. Citizens who perceive high levels of corruption are less likely to prioritize the norm of helping less privileged people in their own country (domestic solidarity).
2. Citizens who perceive high levels of corruption are less likely to prioritize the norm of helping less privileged people in the rest of the world (global solidarity).

However, based on observations made in previous studies there are also several reasons to expect that the individual-level association between corruption perceptions and solidarity norms might vary depending on context. Although previous studies have indicated that corruption perceptions are in some cases negatively associated with support for welfare and redistribution policies (Bauhr and Charron, 2020; Svallfors, 2013), it is not necessarily the case that those who perceive widespread corruption are less willing to help those who are worse off. It might rather be that citizens lack confidence in the government’s ability to implement these policies or that other people would refrain from abusing them. Svallfors (2013), for instance, has shown that in a context of low institutional quality even those citizens who claim that they prefer more economic equality also share a preference for lower taxes and less social spending. Hence, they might believe that it is up to the citizens themselves and the private sector to provide support to the less privileged. In other words, solidarity norms might be relatively strong in a high-corruption context while support for government action is low. Meanwhile, in a context of high institutional quality, respondents of the same “ideological type” prefer more taxes and more social spending (Svallfors, 2013). Thusly, in these contexts we may also see a “crowding out effect” where citizens tend to push the responsibility of helping those who are worse off over to the government and the public welfare sector (Kotzian 2014: 66). Solidarity norms and individual charity might therefore be perceived as less salient here.

Moreover, because corruption is argued to exacerbate existing inequalities and disproportionally harm groups that are socioeconomically disadvantaged due to their dependency on (high quality) public services (Gupta et al. 2002; Justesen and Bjørnskov, 2014; Maeda and Ziegfeld, 2015; Uslaner, 2008), we might also expect to find a stronger emphasis on “asymmetrical charity-based solidarity” (Ahola-Launonen 2019: 183) in contexts where public services are plagued by inefficiency and corruption. The vast inequalities that are often highly visible in these contexts may generate strong feelings of sympathy for those who are often excluded from public services such as healthcare due to their inability to pay bribes or else have to pay relatively large proportions of their meagre earnings.

Furthermore, it might still be the case that citizens demand strong action on the government’s part in reducing domestic inequality, even though they view corruption as an endemic problem and lack trust in the state’s capabilities. Morgan and Kelly (2010), in their study of public attitudes regarding the state’s role in reducing inequalities in 22 Latin American countries, show that perceptions of crime and corruption are positively correlated with greater support for redistribution. The authors argue that citizens are likely to be aware of the previously mentioned connection between corruption and inequality (Uslaner, 2008) and might therefore also be more willing to favor public policies aiming to reduce inequalities if they perceive corruption as a problem (i.e., reducing inequality reduces corruption and vice versa). In the case of global solidarity, moreover, one could also argue for a positive association based on the so-called *compensation hypothesis* and the practice of benchmarking (Bauhr and Charron, 2020). People who are critical of domestic institutions may express greater support for international redistribution and aid due to the fact that they have a higher confidence in the abilities of certain foreign or supranational institutions, such as the EU, who “can be perceived as able to compensate for domestic deficiencies” (Bauhr and Charron 2020: 3). Alternatively, citizens might also be more willing to support international redistribution (and hence show greater global solidarity) as a symbolic protest against the domestic situation or as a result of strategic calculations where they assume that it will benefit other corrupt regions too and improve their own public service quality in the long term.

In contexts where the quality of government is generally judged to be relatively high, in turn, there are reasons to expect those who perceive high levels of corruption to express weaker solidarity. Recent studies argue that perceptions of corruption in relatively well-performing contexts are more likely to express political alienation and anti-establishment sentiments rather than a critique against the current domestic regime and its policies, which is more likely to be the case in more poorly performing contexts (Bauhr and Charron, 2020; Van de Walle, 2008). Recall that the nature of corruption is argued to vary across different contexts (Johnston, 2005), and that affluent “Influence market” societies tend to have more ambiguous forms of corruption centered around lobbying and conflict of interest that are practically invisible to non-elite citizens. Politically alienated persons might be quicker to interpret every “shady” or, using Warren’s (2006) preferred term, “duplicitous” action of established political representatives as corrupt.

This argument is also supported by findings showing that, while it is the less wealthy, the less educated, the less informed and those perceiving unfair treatment on behalf of street-level bureaucrats who tend to perceive corruption as more frequent, these associations mainly hold for the highly developed and relatively equal countries where petty corruption is quite rare (Ariely and Uslaner, 2017; Blais et al. 2017; Maeda and Ziegfeld, 2015). In less developed and more unequal countries, these associations are considerably weaker to the point of sometimes running in the opposite direction (Maeda and Ziegfeld, 2015). In other words, it is likely to be the highly distrustful and dissatisfied “losers of globalization” who tend to hold populist sentiments and perceive widespread corruption among elites in otherwise relatively well performing countries (Akkerman et al. 2014). Recent empirical evidence in support of this potential moderating effect of public sector quality is provided by Bauhr and Charron (2020), who show that perceptions of domestic corruption are positively related to support for within-EU redistribution, but only in contexts of high public sector corruption and low efficiency. They find no such link in contexts of high quality government.

Furthermore, adapting the argument of Morgan and Kelly (2010: 85) concerning crime, one could argue that citizens in wealthy well-functioning states are less likely to connect perceptions of corruption to concrete societal problems than are citizens in poorer countries with highly corrupt and inefficient public services. In other words, citizens are less likely to draw the connection between corruption and inequality, and therefore less likely to become more supportive of redistribution. Hence, the following two hypotheses are formed:

Hypothesis 2a: Citizens who perceive high levels of corruption in high (low) performing contexts are less (more) likely to prioritize the norm of helping less privileged people in their own country (domestic solidarity).
Hypothesis 2b: Citizens who perceive high levels of corruption in high (low) performing contexts are less (more) likely to prioritize the norm of helping less privileged people in the rest of the world (global solidarity).

While there is quite a high correlation between domestic and global solidarity (Pearson’s r = 0.613), there are good reasons why we should examine them as two separate dependent variables. Firstly, as we will see in my final hypothesis, people are generally expected to feel greater identification with and solidarity toward other people who are closer to themselves, both spatially and culturally, and be more supportive of domestic rather than international redistribution (Goodin, 1998; Noël and Thérien, 2002). Although it is said that we live in the age of globalism, where the distance between citizens of different nations has shrunk considerably due to technological, political, and socio-economic developments that have enabled cross-border cooperation in an ever-growing pace, people are still more likely to identify themselves as national citizens rather than global citizens (cosmopolitans), at least in the wealthier countries. For instance, according to a BBC World Service poll conducted by GlobeScan in 2016, only 30% of German respondents perceive themselves as global citizens, compared to 67% in India (Grimley, 2016). Moreover, while there seems to have been a rising trend in “global citizenship” in emerging economies, the trend seems to have been the opposite in wealthy nations after the financial crisis in 2008.

Secondly, as observed by Noël and Thérien (2002: 649), “[p]ublic opinion on international redistribution is not a simple extension of public attitudes about domestic redistribution[…, o]n the contrary, when the two issues are associated at the aggregate level, the relationship is significant but negative.” The authors explain this by pointing at differences in the successful reduction in domestic income disparities in various donor countries. Citizens in countries that historically have been more successful at reducing income disparities, such as Denmark, are argued to view poverty abroad as a more salient problem than poverty at home, while the situation is the opposite in countries such as France where poverty at home has remained a highly salient issue (*Ibid*, 649).

Thirdly, agreeing that people generally can be trusted does not necessarily mean that people generally trust other people independent of the context where they are located (Rothstein and Eek, 2009). Showing blind trust toward people from contexts characterized by endemic corruption can hence be considered naïve. As research on foreign aid to developing countries has shown (Bauhr et al. 2013; Chong and Gradstein 2008; Paxton and Knack 2012), foreign aid is still a controversial issue with varying levels of “aid fatigue” among citizens and actual amounts of aid transfers (relative to GDP) among various donor countries. This is reflected for instance in the calculations of Kopczuk et al. (2005)in Chong and Gradstein 2008: 2), showing “that actual levels of foreign aid relative to domestic income transfers correspond to citizens in rich countries attaching the welfare of citizens in poor countries only 1/2000 of the weight assigned to the welfare of their own poor.” Meagre results and corruption in the recipient countries are often held to be some of the main reasons behind aid fatigue (see e.g., Bauhr et al. 2013: 569); however, studies have also pointed at perceptions of domestic corruption and inefficiencies in the donor countries (Bauhr et al. 2013; Bodenstein and Faust, 2017; Chong and Gradstein, 2008). The latter finding is argued to be explained by the fact that perceptions of wasted or misused tax money by domestic institutions act as a proxy for trust in the country’s development assistance and its chances for success, thereby resulting in a spillover effect.

Fourthly, in addition to antiglobalism, *nativism*^4^ is also often considered an important component of radical right-wing populism, which in turn is closely connected to the earlier discussed anti-establishment sentiments (Betz 2019; Heinrich et al. 2019; Mudde and Rovira Kaltwasser 2012; Verbeek and Zaslove 2017). Hence, it is likely that people who hold these kinds of attitudes are considerably more willing to help their own fellow (native-born) compatriots rather than foreigners or immigrants.

For the purposes of this study, it is especially relevant to look closer at the results of Bodenstein and Faust (2017), who analyzed Eurobarometer data of 27 EU Member States and found that individual corruption perceptions are negatively related to support for foreign aid in 15 traditional EU donor countries and positively related in 12 new EU donor countries. Although they do not elaborate more on this finding (they only note that the effect of corruption perceptions, which was negative but non-significant for the whole sample of EU donor states, remain robust for traditional donor countries), this finding would seem to provide some support for my previous hypotheses, at least with regard to global solidarity.

Combining these arguments and observations, I expect that global solidarity is generally lower than domestic solidarity. Furthermore, I expect the negative effects of corruption perceptions to be considerably stronger for global solidarity. Hence, the following hypothesis is formed:

Hypothesis 3: The negative association between corruption perceptions and solidarity with the less privileged is stronger for global solidarity, which tends to be generally lower than domestic solidarity.

## Data, Variables, and Methods

**Fig. 1:**
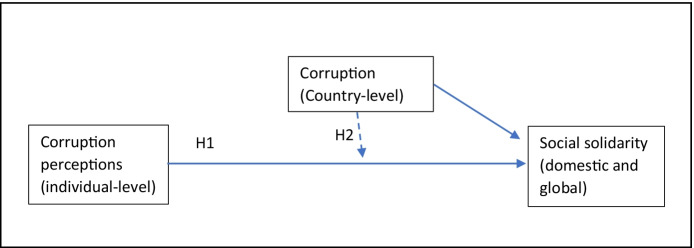
Research model

The research model is described in Fig. 1, where the solid arrow “H1” represents the hypothesized (negative) association between individual corruption perceptions and the two dependent variables. The second solid arrow and the dashed arrow “H2” meanwhile represent the potential contextual and moderating effects of the country-level corruption context.

The data originate primarily from the International Social Survey Programme (ISSP) and the Quality of Government Institute (QoG), in addition to a few other sources (see variable descriptions below). The individual-level data come from ISSP study Citizenship II, covering 33 countries (ISSP 2016).^5^ This dataset is ideal for the purposes since it contains questions regarding corruption perceptions and solidarity norms concerning support for the less privileged both domestically and globally.^6^ Next, I present information on key variables (For detailed information on question wordings and coding of variables, see Appendix B).

*Domestic and global solidarity*: The two dependent variables in this study are constructed using the following two questions from the ISSP Citizenship II survey: *There are different opinions as to what it takes to be a good citizen. As far as you are concerned personally on a scale of 1 to 7, where 1 is not at all important and 7 is very important, how important is it:* [1] *To help people in (COUNTRY) who are worse off than yourself* (domestic solidarity); [2] *To help people in the rest of the world who are worse off than yourself* (global solidarity). The variables are recoded to range from 0 to 1 (1 = highest level of solidarity).

*Corruption perceptions*: The independent variable of the study is *individual* c*orruption perceptions (ICP)*. This is operationalized using one of the ISSP items asking respondents “*How widespread do you think corruption is in the public service in (COUNTRY)?”* Response alternatives are (1) Hardly anyone involved; (2) A small number of people are involved; (3) A moderate number of people are involved; (4) A lot of people are involved; (5) Almost everyone is involved. The variable is recoded to range from 0 to 1 (1 = highest level of perceived corruption).

At the macrolevel, this study operationalizes *country-level corruption perception* (CCP) with Transparency International’s Corruption Perceptions Index (CPI). This is the most widely used indicator of corruption worldwide and has been compiled on a yearly basis since 1995. Although this corruption indicator is also perceptions based and has its own share of problems (see e.g., Andersson and Heywood 2009), it is based on the perceptions of international experts and businesspeople rather than domestic citizens. Hence, it is arguably more objective and useful when comparing different kinds of corruption contexts than a measure that simply aggregates individual corruption perceptions such as those in the ISSP. It uses (since 2012) a scale of 0 to 100, where 0 is highly corrupt and 100 is very clean. Here, the scale has been reversed and recoded so that it ranges from 0 (very clean) to 1 (highly corrupt).

To ascertain the associations found here, I include several control variables that have been known to influence social cohesion and support for domestic and foreign redistribution, and therefore may confound the results (Bayram, 2017; Bodenstein and Faust 2017; Kotzian 2014; Paxton and Knack 2012). At the individual level, I include standard socio-demographic characteristics age (measured in age groups 1–6, each covering 15 years), gender (male or female), education (years of education completed), marital status (married or not), place of living (urban or rural), and employment status (unemployed or other). I also include several individual factors that may influence the propensity for social solidarity since this is customary in the literature on civic norms (see e.g., Kotzian 2014).^7^ These include indicators of religiosity (frequency of attendance at religious services), political self-placement (left/right), generalized trust, and political interest (no interest at all–very interested).

I also include several country-level control variables to control for contextual differences. While it is impossible to include all possible aspects, I chose to control for theoretically relevant aspects that previous studies suggest might affect domestic and global solidarity (Ariely 2014; Kotzian, 2014; Putnam 2007; Rothstein and Uslaner 2005). These include, among other things, *level of inequality* (Gini; V-Dem, 2018; UNU-WIDER, 2017) and *ethnic fractionalization* (Alesina et al. 2003; QoG, 2020).^8^ All data for the macrovariables concern the years 2012–13, except for the measures of fractionalization, which concern the year 2000.

All variables except age and education are coded to vary between 0 and 1 to make the results more comparable. Table 1 contains summary information on all variables.

**Table 1:** Descriptive Statistics

	Obs	Mean	SD	Min	Max
Dependent variables					
Domestic solidarity	47,602	0.77	0.24	0.00	1.00
Global solidarity	47,124	0.64	0.30	0.00	1.00
Independent variable					
Individual corruption perception (ICP)	43,965	0.51	0.27	0.00	1.00
Moderating variable					
Country-level corruption perception (CCP)	48,800	0.39	0.25	0.00	1.00
Control variables					
Gender (male)	48,776	0.47	0.50	0.00	1.00
Age group	48,662	2.74	1.21	1	6
Education	47,464	12.4	4.09	0	30
Marriage status (married)	48,347	0.53	0.50	0.00	1.00
Place of living (urban)	48,293	0.69	0.46	0.00	1.00
Employment status (unemployed)	48,153	0.08	0.27	0.00	1.00
Generalized trust	47,043	0.44	0.26	0.00	1.00
Political interest	47,541	0.47	0.30	0.00	1.00
Religious attendance	46,711	0.37	0.33	0.00	1.00
Political self-placement (Left/Right)	39,934	0.53	0.24	0.00	1.00
Ethnic fractionalization	48,800	0.36	0.27	0.00	1.00
Economic inequality (Gini)	48,800	0.28	0.26	0.00	1.00

This study utilizes multilevel linear regression (OLS) analyses in STATA 16.0 to take into account that respondents are nested in countries. Since the dataset does not include comparable weights across countries, I use unweighted data, which mean that the results are not necessarily representative of the target populations in each country.

## Empirical Analyses

I begin the analyses by in Table 2 presenting country-level differences in solidarity norms and corruption perceptions.

**Table 2:** Country-Level Differences

	Domestic solidarity		Global solidarity		Individual corruption perception		Country-level corruption perception
	Mean	SE	Mean	SE	Mean	SE	Score
AU-Australia	0.80	0.01	0.60	0.01	0.39	0.01	0.14
AT-Austria	0.81	0.01	0.61	0.01	0.45	0.01	0.31
BE-Belgium	0.68	0.01	0.57	0.01	0.40	0.01	0.23
CL-Chile	0.87	0.01	0.79	0.01	0.62	0.01	0.28
TW-Taiwan	0.79	0.01	0.63	0.01	0.50	0.01	0.42
HR-Croatia	0.85	0.01	0.74	0.01	0.67	0.01	0.61
CZ-Czech Republic	0.66	0.01	0.56	0.01	0.64	0.01	0.61
DK-Denmark	0.74	0.01	0.60	0.01	0.25	0.01	0.00
FN-Finland	0.71	0.01	0.51	0.01	0.32	0.01	0.03
FR-France	0.70	0.01	0.57	0.01	0.46	0.01	0.28
GE-Georgia	0.93	0.01	0.75	0.01	0.28	0.01	0.59
DE-Germany	0.74	0.01	0.60	0.01	0.43	0.01	0.18
IS-Iceland	0.81	0.01	0.65	0.01	0.49	0.01	0.18
IN-India	0.74	0.01	0.62	0.01	0.55	0.01	0.77
IL-Israel	0.81	0.01	0.54	0.01	0.61	0.01	0.42
JP-Japan	0.66	0.01	0.61	0.01	0.47	0.01	0.24
KR-Korea (South)	0.70	0.01	0.56	0.01	0.57	0.01	0.51
LT-Lithuania	0.77	0.01	0.56	0.01	0.68	0.01	0.48
NL-Netherlands	0.75	0.01	0. 65	0.01	0.31	0.01	0.11
NO-Norway	0.76	0.01	0.70	0.01	0.33	0.01	0.07
PH-Philippines	0.86	0.01	0.77	0.01	0.62	0.01	0.77
PL-Poland	0.72	0.01	0.60	0.01	0.63	0.01	0.44
RU-Russia	0.65	0.01	0.52	0.01	0.67	0.01	0.89
SK-Slovak Republic	0.69	0.01	0.59	0.01	0.63	0.01	0.62
SI-Slovenia	0.84	0.01	0.66	0.01	0.66	0.01	0.48
ZA-South Africa	0.77	0.01	0.70	0.01	0.65	0.01	0.69
ES-Spain	0.88	0.01	0.79	0.01	0.55	0.01	0.45
SE-Sweden	0.72	0.01	0.66	0.01	0.37	0.01	0.03
CH-Switzerland	0.77	0.01	0.67	0.01	0.35	0.01	0.08
TR-Turkey	0.89	0.01	0.83	0.01	0.60	0.01	0.58
GB-Great Britain	0.73	0.01	0.57	0.01	0.37	0.01	0.21
US-United States	0.81	0.01	0.61	0.01	0.55	0.01	0.25
VE-Venezuela	0.92	0.01	0.85	0.01	0.75	0.01	1.00
Mean	0.77	0.01	0.64	0.01	0.51	0.01	0.39

As we can see from the table, there is considerable variation in people’s expressed willingness to help those who are worse off, both with regard to the sphere of solidarity (domestic or global) and to the society in question. For domestic solidarity, we can see that, as expected, it is on average higher than global solidarity (0.77 > 0.64). Here, in the top three highest average scores we find such countries as Georgia (0.93), Venezuela (0.92), and Turkey (0.89), while in the bottom three we find countries like Russia (0.65), the Czech Republic (0.66), and Japan (0.66). For global solidarity, we find a similar pattern with Venezuela (0.85), Turkey (0.83), and Chile/Spain (0.79) in the top and Finland (0.51), Russia (0.52), and Israel (0.54) in the bottom. Furthermore, we can also note that there is not a single case where the average global solidarity is higher than the average domestic solidarity. The largest gap between the two solidarity norms can be found in Israel (0.81 & 0.54), while the smallest can be found in Japan (0.66 & 0.61).

We can also observe clear differences across countries for the two corruption indicators; however, the most interesting observation is possibly that the subjective individual perceptions do not appear to be clearly linked to the expert observations at the country level. Citizens generally appear to have a higher tendency to think that public services are corrupt, with some notable exceptions such as India, which receives the highest corruption score by the experts while citizens believe that it is relatively low (0.55).

Table 3 meanwhile includes the results of multilevel linear regression analyses for domestic and global solidarity. Here, model 1 (M1) is fixed effects bivariate regressions between the dependent variable in question and individual corruption perceptions (ICP), M2 includes all control variables; M3 includes an interaction effect between ICP and CCP as well as a random intercept for ICP. After this, the implications for the hypotheses are discussed using plots of marginal means to elucidate the implications following the recommendations of Brambor et al. (2006).

**Table 3:** Regression Results

	M1	Domestic solidarity M2	M3	M1	Global solidarity M2	M3
Individual corruption perception (ICP)	0.005 (0.005)	0.021*** (0.005)	-0.003 (0.02)	-0.057*** (0.006)	-0.033*** (0.007)	-0.113*** (0.027)
Gender (male)		-0.032*** (0.002)	-0.033*** (0.002)		-0.052*** (0.003)	-0.052*** (0.003)
Age group		-0.002 (0.005)	-0.002 (0.005)		-0.025*** (0.006)	-0.025*** (0.006)
Age group squared		0.001 (0.001)	0.001 (0.001)		0.003** (0.001)	0.003** (0.001)
Education		-0.001 (0.000)	-0.001 (0.000)		0.002** (0.000)	0.001** (0.013)
Marriage status (married)		-0.001 (0.003)	-0.002 (0.003)		0.001 (0.003)	0.000 (0.003)
Place of living (urban)		0.003 (0.003)	0.003 (0.003)		0.008* (0.004)	0.007* (0.004)
Employment status (unemployed)		0.003 (0.005)	0.002 (0.005)		0.000 (0.006)	0.000 (0.006)
Generalized trust		0.030*** (0.005)	0.029*** (0.005)		0.057*** (0.007)	0.056*** (0.007)
Political interest		0.072*** (0.005)	0.074*** (0.005)		0.074*** (0.006)	0.074*** (0.006)
Religious attendance		0.050*** (0.004)	0.049*** (0.004)		0.089*** (0.005)	0.087*** (0.005)
Left/right position		-0.068*** (0.005)	-0.067*** (0.005)		-0.123*** (0.006)	-0.122*** (0.006)
Country corruption perception (CCP)		-0.004 (0.055)	-0.006 (0.057)		0.043 (0.065)	-0.004 (0.065)
Ethnic fractionalization		0.043 (0.061)	0.034 (0.062)		0.022 (0.071)	0.013 (0.071)
Economic inequality (Gini)		0.110 (0.065)	0.082 (0.067)		0.131 (0.077)	0.084 (0.077)
ICP#CCP			0.044 (0.041)			0.164** (0.057)
Constant	0.772*** (0.013)	0.710*** (0.026)	0.725*** (0.026)	0.672*** (0.016)	0.610*** (0.030)	0.651*** (0.030)
Random effects: Var (Cons)	0.005*** (0.001)	0.005*** (0.001)	0.005*** (0.001)	0.008*** (0.002)	0.006*** (0.002)	0.006*** (0.002)
Var(residual)	0.052*** (0.000)	0.049*** (0.000)	0.049*** (0.000)	0.080*** (0.001)	0.075*** (0.001)	0.075*** (0.001)
Var(ICP)			0.003*** (0.001)			0.006*** (0.002)
Obs. (groups)	43,128(33)	33,152(33)	33,152(33)	42,792(33)	32,969(33)	32,969(33)
BIC	-4528.98	-5238.75	-5279.75	13,562.29	8538.75	8432.73
ICC	0.094	0.086	0.087	0.093	0.079	0.076

Entries are coefficients from a multilevel linear regression with standard errors in parenthesis. *** *p* < 0.001, ** *p* < 0.01, * *p* < 0.05

Contrary to the expectation that there would be a negative association between perceptions of corruption and domestic solidarity, I find a *positive*, although insignificant, coefficient in M1 (B = 0.005 *p* = 0.261), suggesting that corruption perceptions *increase* domestic solidarity. Furthermore, the coefficient grows highly significant when including controls in M2 (B = 0.021, *p* = 0.000). I thereby find *no support for H1a*.

**Fig. 2:**
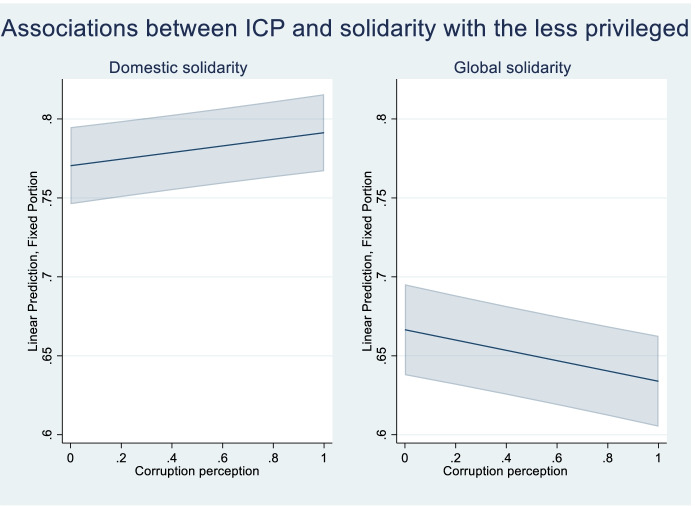
Estimated average associations between ICP and social solidarity (95% CI)

For global solidarity, in turn, I observe the expected *negative* association in M1 (B = -0.057 *p* = 0.000), indicating that global solidarity *declines* as people perceive more corruption in the public services. After including controls in M2, the coefficient shrinks by almost half, but remains highly significant (B = -0.033 *p* = 0.000). Contrary to the case for domestic solidarity, then, I find relatively *strong support for H1b*. Curiously then, it would appear that corruption perceptions have opposite effects depending on the type of solidarity. Figure 2 displays the estimated effect of individual corruption perception on domestic and global solidarity without taking into account contextual variations, but controlling for other factors obtained in M2.

**Fig. 3:**
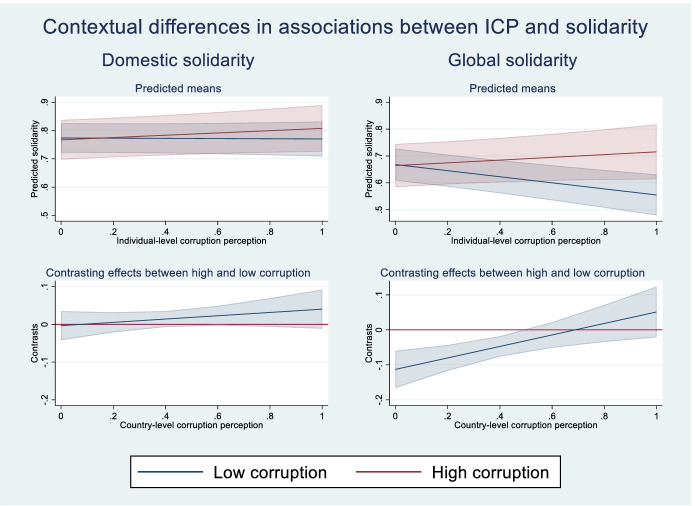
Contextual differences in the estimated associations between ICP and predicted solidarity (95% CI)

From the left side plot, we can see that there is an increase in the predicted strength of domestic solidarity norms as the individual perception of corruption increases, but the estimate is surrounded by uncertainty as indicated by the wide confidence intervals. The association entails that the expected strength of domestic solidarity norms increases from 0.77 to 0.79 as ICP moves from minimum to maximum. From the right side plot, in turn, we can see that the expected strength of global solidarity norms decreases from 0.67 to 0.64 as ICP moves from minimum to maximum. It is worth noting that, while the estimates themselves are relatively weak on average, they have quite broad confidence intervals, indicating that there could be important differences between individuals in different contexts, as posited by my hypotheses. Next, I demonstrate the contextual differences across various levels of macrocorruption obtained in M3 in Fig. 3.

The left-hand panels show the predicted means and contrasting effects for domestic solidarity across different CPI-values, while the right-hand ones show the same thing for global solidarity. For domestic solidarity, the insignificant interaction effect in M3 (B = 0.044, *p* = 0.280) entails that there are no significant differences in the effect of individual perceptions of corruption on predicted domestic solidarity across different levels of institutional quality. The positive sign of the coefficient would suggest, as we can see from the plot in the upper left side corner, that there is a positive effect in highly corrupt environments and no effect in highly “clean” ones. However, due to the lack of statistical significance we can draw no firm conclusions. Hence, I find *no support for H2a*.

Turning to global solidarity, I find a strong and highly significant *positive* interaction effect (B = 0.164, *p* = 0.004) in M3. This implies that, as seen in the upper right side plot, there is a comparatively weaker *increase* in the predicted strength of global solidarity norms as the individual perception of corruption increases in highly corrupt contexts (from 0.67 when ICP = 0.0 to 0.72 when ICP = 1.0) and a relatively strong *decline* in the predicted strength of global solidarity norms as the individual perception of corruption increases in less corrupt contexts (from 0.67 when ICP = 0.0 to 0.56 when ICP = 1.0). Therefore, these results provide *strong support for H2b*, especially in the case of low corruption contexts where perceptions of corruption in the public services have a strong negative effect on solidarity with the less privileged in the rest of the world.

As we can see when we compare the models for domestic solidarity on one hand and global solidarity on the other, the (negative) effects are clearly and consistently larger for global solidarity, which generally tends to be lower than domestic solidarity. Hence, the results provide *strong support for H3*.

Finally, the results for the control variables are largely as expected based on earlier studies, at least for the individual-level ones. Women, those with a high generalized trust and political interest, those who frequently attend religious services, and those who position themselves on the political left tend to put a significantly higher priority on domestic solidarity. Meanwhile, women, the youngest and oldest age groups, the highly educated, urban residents, those with a high generalized trust and political interest, those who frequently attend religious services, and those who position themselves on the political left tend to put a significantly higher priority on global solidarity. Political self-placement, religiosity, and political interest seem to be among the strongest predictors in both cases, while age group (non-linear) is a very strong predictor for global solidarity. None of the country-level controls show any significance.^9^

### Robustness Checks

**Fig. 4:**
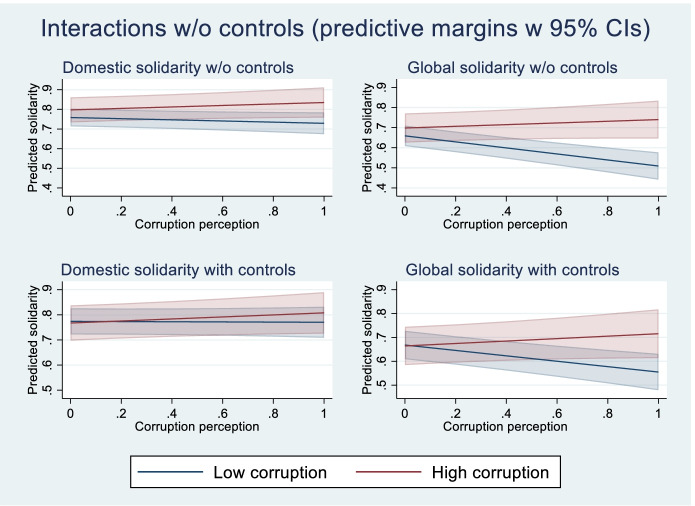
Model specification

**Fig. 5:**
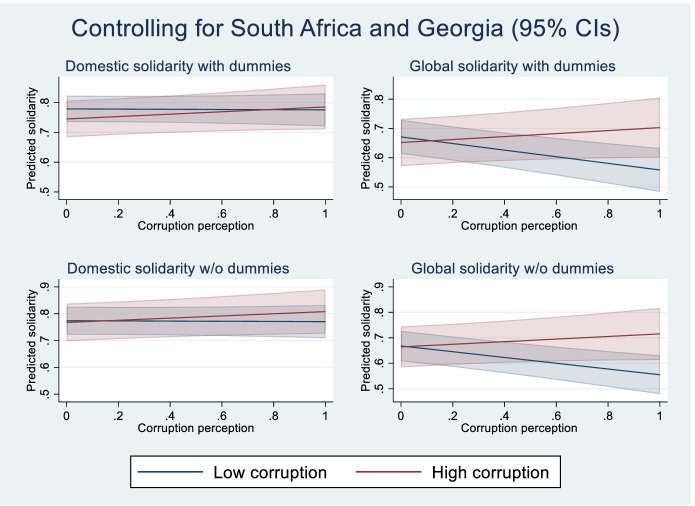
Controlling for influential cases

**Fig. 6:**
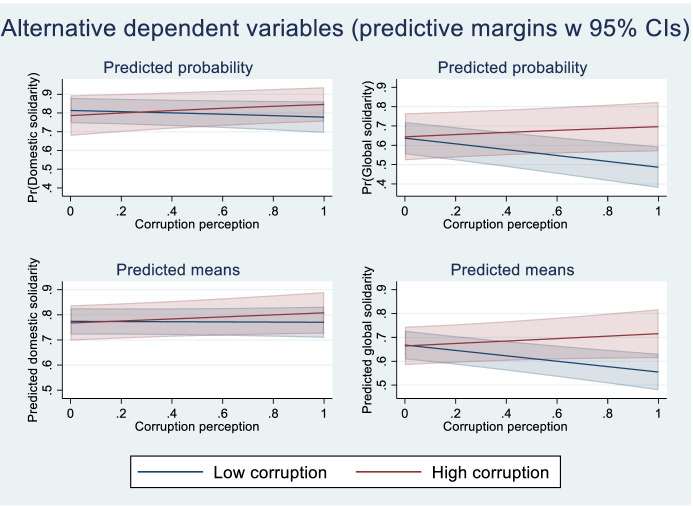
Logistic regression (ml) with dichotomous versions of dependent variables (1/4 = 0, 5/7 = 1)

Further analyses reveal that the models are robust to different specifications (see Fig. 4 in Appendix A). The models are still robust after the inclusion of dummy variables for two influential outliers, South Africa and Georgia (see Fig. 5). I also re-run my models using logistic multilevel regression and alternative versions of my dependent variables where the answer alternatives in the two survey items are collapsed into binary choices^10^ (see Fig. 6). The results (not shown but available upon request) for global solidarity are substantially similar to my main results. The predicted probability of viewing global solidarity as important grows from approximately 0.64 to 0.70 in a high corruption context, while it declines from 0.64 to 0.49 in a low corruption context as individual corruption perceptions increase. For domestic solidarity, on the other hand, the results show a borderline significant interaction term (B = 0.685, *p* = 0.055).

## Discussion and Conclusions

This study sought out to acquire deeper understanding of the associations between individual perceptions of corruption, a factor often argued to be a strong determinant of social cohesion and social solidarity both on a domestic and on a global plane. To my knowledge, it is the first of its kind to examine cross-level variations in the association between individual perceptions of corruption and two different types of social solidarity. More precisely, I argued that there are strong reasons to expect these associations to vary across different societal contexts depending on the generally perceived quality of public institutions. To test this, I used data from the ISSP Citizenship II survey, covering 33 countries.

The study finds strong empirical support for the hypothesis concerning the differences in effects between domestic and global solidarity (H3) where I observe considerably stronger effects for global solidarity, which generally tends to be lower than domestic solidarity. Additionally, I observe strong support for my hypotheses regarding global solidarity (H1b & H2b). Holding all else equal, I find a highly significant negative relationship between individual corruption perceptions and solidarity with those who are worse off in the rest of the world. However, this strong negative association seems to be primarily driven by citizens living in highly developed countries that according to TI’s Corruption Perceptions Index are relatively good at keeping corruption (or at least some forms of it) in check. In highly corrupt countries, in turn, there is a somewhat weaker positive association between these two variables. For domestic solidarity, on the other hand, I find no significant support for neither H1a nor H2a. Initial analysis reveals a weak positive association between corruption perceptions and domestic solidarity, while cross-level analysis suggests that this link can principally be found in high-corruption contexts. But there is no evidence of a significant negative association between these two variables in any of the examined contexts.

In sum, perceptions of corruption in the public services mainly seem to matter in the case of solidarity norms concerning less privileged people beyond domestic borders. Moreover, there are obvious differences across highly “clean” and highly corrupt contexts where we can clearly see two opposite effects. In the case of domestic solidarity, individual corruption perceptions seem to matter very little, if at all.

These results are in line with the conclusion in Bauhr and Charron (2020) that it is essential to look beyond the average effects of individual determinants of social solidarity and assumptions of homogenous effects and causal mechanisms. Ignoring contextual variations, as many previous studies have done, could thereby result in highly misleading conclusions. Furthermore, these results are generally in line with what one would expect based on the observation made by Van de Walle (2008) and Bauhr and Charron (2020), among others, that perceptions of widespread corruption in high-performing contexts are likely to be a symptom of anti-establishment (populist) attitudes and a frustration with the current state of democracy (Zaslove et al. 2020). These “disappointed democrats” are likely to be suspicious and critical of endeavors that channel resources abroad (potentially in the pockets of corrupt elites) instead of being used to aid the less fortunate at home (e.g., “America First”). Inglehart and Norris 2016: 7), for instance, observe that “[p]opulism favors […] national self-interest over international cooperation and development aid” (also see Heinrich et al. 2019; Jakupec and Kelly 2019; Verbeek and Zaslove 2017).

Similarly, the weaker positive association between corruption perceptions and global solidarity that can be found in contexts generally perceived as more corrupt resembles the findings in Bauhr and Charron (2020) regarding support for EU-wide redistribution. The reason for this could potentially be that citizens show a higher trust in foreign donor states’ or international organizations’ capabilities in managing these types of redistributions and/or that they count on benefiting from a higher global solidarity, at least in the long term. Additionally, they might be better positioned to draw the connection between (global) inequality and corruption, as argued by Morgan and Kelly (2010).

The lack of any significant effects of corruption perceptions on domestic solidarity in turn suggests that there are other mechanisms at play than those conjectured based on the observed association between corruption perceptions and social trust. While perceived corruption might reduce social trust, it does not seem to be related to the stated willingness to help fellow compatriots who are worse off. Other factors, such as cultural, ideological, and religious norms and values, or the perceived saliency of solidarity norms, probably play a much larger role than perceived institutional quality.

However, caution is needed when interpreting these results and their policy implications. Firstly, due to the unfortunately vague question wordings of the dependent variables we do not know exactly what kind of “help” respondents are thinking of. For instance, is it humanitarian aid (e.g., food and medicine) or development aid (e.g., investments in infrastructure and education)? Is it mainly the state’s or a non-state actor’s responsibility to provide aid to less privileged people? The question wordings could point at the responsibility of ordinary (good) citizens. However, the costliness of “asymmetrical charity-based solidarity” (Ahola-Launonen 2019: 183) and its low efficacy (due to the free rider problem) would in turn suggest that the average respondent is more likely to view it largely as the state’s responsibility, while at the same time being more willing to provide resources through taxation. The results of Morgan and Kelly’s (2010) study, which showed some similarities with the results in this one regarding the sometimes positive effects of corruption perceptions on (domestic) solidarity, would seem to support this interpretation, due to the fact that they specifically asked about the role of the state in redistribution. Still, we cannot rule out that citizens in societies with poorly performing public institutions are more likely to prefer that non-state actors such as labor unions, churches, private charity and/or inter/supranational organizations shoulder the main responsibility in delivering aid to the poor (Svallfors 2013). This could potentially explain why we sometimes see a positive association between corruption perceptions and social solidarity in highly corrupt contexts. Future studies of solidarity norms would have to include more detailed questions in order to bring greater clarity to this issue.

Secondly, the results may be plagued by social desirability bias (SDB), which means that respondents could be indicating an unnaturally high support for solidarity norms in an attempt to be socially pleasing (Tourangeau and Yan 2007). This problem could further be compounded by cultural differences in the prevalence and strength of SDB. Thirdly, due to the cross-sectional nature of this study, I cannot draw any firm conclusions regarding causality. We would require panel data or experimental studies to say if perceptions of corruption influence solidarity norms or vice versa. Experimental studies would also be useful in minimizing the problem of SDB. In short, more research is clearly needed within this important and highly topical field.

## Appendix

Appendix A: Robustness checks

Please see Figs. 4, 5 and 6.

Appendix B: Question wording

Please see Table

**Table 4:** Question Wording

Item	Question	Answer alternatives
Q8 Good citizen: help less privileged people in (COUNTRY) [domestic solidarity]	There are different opinions as to what it takes to be a good citizen. As far as you are concerned personally on a scale of 1 to 7, where 1 is not at all important and 7 is very important, how important is it: To help people in [COUNTRY] who are worse off than yourself	(1) Not at all important (7) Very important (8) Can’t choose (9) No answer
Q9 Good citizen: help less privileged people in the rest of world [global solidarity]	There are different opinions as to what it takes to be a good citizen. As far as you are concerned personally on a scale of 1 to 7, where 1 is not at all important and 7 is very important, how important is it: To help people in the rest of the world who are worse off than yourself	(1) Not at all important (7) Very important (8) Can’t choose (9) No answer
Q67 Public service: involvement in corruption [individual corruption perception, ICP]	How widespread do you think corruption is in the public service in [COUNTRY]?	(1) Hardly anyone is involved (2) A small number is involved (3) A moderate number is involved (4) A lot of people are involved (5) Almost everyone is involved (8) Can’t choose (9) No answer
BVQ_01. SEX [gender]	Sex of respondent	(19 Male (2) Female (9) No answer
BVQ_02a. AGE	Age of respondent	(15) 15 years (102) 102 years (999) No answer
BVQ_03. EDUCYRS [education]	How many full years of schooling or education have you had? Please include primary and secondary schooling, university and full-time vocational training, but do not include repeated years	(0) No formal schooling, no years at school (1) One year (83) 83 years (98) Don’t know (99) No answer
BVQ_34. MARITAL [marriage status]	What is your current legal marital status?	(1) Married (2) Civil partnership (3) Separated from spouse/civil partner (still legally married/still legally in a civil partnership) (4) Divorced from spouse/legally separated from civil partner (5) Widowed/civil partner died (6) Never married/never in a civil partnership, single (7) Refused (9) No answer
BVQ_37. URBRURAL [place of living]	Would you describe the place where you live as…	(1) A big city (2) The suburbs or outskirts of a big city (3) A town or a small city (4) A country village (5) A farm or home in the country (8) Don’t know (9) No answer
BVQ_14. MAINSTAT [employment status]	Which of the following best describes your current situation? If you temporarily are not working because of temporary illness/ parental leave/ vacation/ strike etc., please refer to your normal work situation	(1) In paid work (2) Unemployed and looking for a job (3) In education (4) Apprentice or trainee (5) Permanently sick or disabled (6) Retired (7) Domestic work (8) In compulsory military service or community service (9) Other (99) No answer
Q48 People can be trusted or can't be too careful in dealing with? [generalized trust]	Generally speaking, would you say that people can be trusted or that you can't be too careful in dealing with people?	(1) People can almost always be trusted (2) People can usually be trusted (3) You usually can't be too careful in dealing with people (4) You almost always can't be too careful in dealing with people (8) Can’t choose (9) No answer
Q43 Level of personal interest in politics [political interest]	How interested would you say you personally are in politics?	(1) Very interested (5) Not at all interested (8) Can’t choose (9) No answer
BVQ_24 ATTEND: Attendance of religious services [religious attendance]	Apart from such special occasions as weddings, funerals, etc., how often do you attend religious services?	(1) Several times a week or more often (incl. every day, several times a day) (2) Once a week (3) 2 or 3 times a month (4) Once a month (5) Several times a year (6) Once a year (7) Less frequently than once a year (8) Never (97) Refused (98) Don’t know (99) No answer
Q44 Political self-placement: left–right [left/right position]	In politics people sometimes talk of left and right. Where would you place yourself on a scale from 0 to 10 where 0 means the left and 10 means the right?	(0) Left (10) Right (98) Can’t choose (99) No answer

4.

## Footnotes

1 [1] Collective good, [2] distribution, [3] support, and [4] loyalty norms (Tranow 2019: 40).

2 This, as pointed out by Tranow (2019: 44), is also quite close to the everyday understanding of the concept of solidarity.

3 Kotzian (2014: 63) also points out the potential costliness of this norm in that it “demands immediate personal action, not just abstaining from doing something[…, i]f taken seriously, the norm is also costly in demanding substantial personal effort, in both financial and nonfinancial terms”.

4 Defined by Betz (2019: 2) as “an expressed partiality to the native-born and their culture in preference to the foreign-born”.

5 Hungary is excluded because of missing data for all respondents on years of education. Sensitivity analyses revealed that South Africa and Georgia constitute outliers in at least some of the models. I therefore reran all models including dummy variables for South Africa and Georgia, but this did not substantively affect the main results.

6 Due to reasons of data availability, this sample of 33 countries is mainly restricted to high- and middle-income countries. Hence, it is difficult to surmise if the results of this study would also extend to low-income countries, although I see no obvious reasons why they would not. Future studies can hopefully shed a light on these questions with more extensive data sets.

7 These attitudes could in turn be affected by corruption perceptions, meaning my results provide a conservative estimate of the impact of individual corruption perceptions since I may remove any indirect effects mediated through these attitudes.

8 I also control for a number of other theoretically relevant macro variables such as *GDP per capita (logged)* (World Bank, 2020) and *public welfare spending* (% of GDP; WHO, 2020). However, due to problems with multicollinearity and/or missing country data I exclude them from my main analyses. Additionally, I control for the *post-communist countries*, due to the special historical and ideological circumstances that scholars argue have shaped social cohesion in these societies (Karklins, 2005). All results are available upon request.

9 Gini comes closest to statistical significance (B = 0.110, *p* = 0.091 for domestic solidarity and B = 0.131, *p* = 0.087 for global solidarity).

10 All respondents who answer “1” (Not at all important) to “4” are coded “0,” while all who answer “5” to “7” (Very important) are coded “1”.

## References

[CR1] Ahola-Launonen J. If solidarity is the answer, what was the question? In: Althammer J, Neumärker B, Nothelle-Wildfeuer U, editors. Solidarity in Open Societies*.* Wiesbaden: Springer VS; 2019. p. 173–90.

[CR2] Akkerman A, Mudde C, Zaslove A (2014). How Populist Are the People? Measuring Populist Attitudes in Voters. Comp Polit Stud.

[CR3] Alesina A, Devleeschauwer A, Easterly W, Kurlat S, Wacziarg R (2003). Fractionalization. J Econ Growth.

[CR4] Almond GA, Verba S (1963). The civic culture: Political attitudes and democracy in five nations.

[CR5] Althammer J. Solidarity: From small communities to global societies. In: Althammer J, Neumärker B, Nothelle-Wildfeuer U, editors. Solidarity in Open Societies. Wiesbaden: Springer VS; 2019. p. 5–23.

[CR6] Andersson S, Heywood PM (2009). The Politics of Perception: Use and Abuse of Transparency International's Approach to Measuring Corruption. Political Stud..

[CR7] Ariely G (2014). Does Diversity Erode Social Cohesion? Conceptual and Methodological Issues. Polit Stud-London.

[CR8] Ariely G, Uslaner E (2017). Corruption, fairness, and inequality. Int Polit Sci Rev.

[CR9] Bauhr M, Charron N, Nasiritousi N (2013). Does Corruption Cause Aid Fatigue? Public Opinion and the Aid-Corruption Paradox. Int Stud Quart.

[CR10] Bauhr M, Charron N (2020). The EU as a savior and a saint? Corruption and public support for redistribution. J Eur Public Policy.

[CR11] Bayram AB (2017). Aiding Strangers: Generalized Trust and the Moral Basis of Public Support for Foreign Development Aid. Foreign Policy Anal.

[CR12] Betz H-G (2019). Facets of nativism: a heuristic exploration. Patterns Prejudice.

[CR13] Bigoni M, Bortolotti S, Casari M, Gambetta D, Pancotto F (2016). Amoral Familism, Social Capital, or Trust? The Behavioural Foundations of the Italian North-South Divide. Econ J.

[CR14] Blais A, Gidengil E, Kilibarda A (2017). Partisanship, Information, and Perceptions of Government Corruption. Int J Public Opin R.

[CR15] Bodenstein T, Faust J (2017). Who Cares? European Public Opinion on Foreign Aid and Political Conditionality. J Common Mark Stud.

[CR16] Brambor T, Clark WR, Golder M (2006). Understanding Interaction Models: Improving Empirical Analyses. Polit Anal.

[CR17] Chong A, Gradstein M (2008). What Determines Foreign Aid? The Donors' Perspective. J Dev Econ.

[CR18] Dalton RJ (2008). Citizenship norms and the expansion of political participation. Polit Stud-London.

[CR19] Denters B, Gabriel O, Torcal M, van Deth JW, Montero R, Westholm A (2007). Norms of Good Citizenship. Citizenship and Involvement Among the Populations of European Democracies.

[CR20] Goodin R (1988). What is So Special about Our Fellow Countrymen?. Ethics.

[CR21] Grimley N. Identity 2016: 'Global citizenship' rising, poll suggests. BBC World Service, April 28, 2016. https://www.bbc.com/news/world-36139904. Accessed 8.6.2020.

[CR22] Gupta S, Hamid D, Alonso-Terme R (2002). Does corruption affect income inequality and poverty?. Econ Gov.

[CR23] Habibov N, Cheung A, Auchynnikava A (2017). Does Social Trust Increase Willingness to Pay Taxes to Improve Public Healthcare? Cross-sectional Cross-country Instrumental Variable Analysis. Soc Sci Med.

[CR24] Hechter M. Principles of Group Solidarity. 1st paperback ed Berkeley: University of California Press; 1988.

[CR25] Heinrich T, Kobayashi Y, Lawson, E Jr. Populism and Foreign Aid. Conference paper 2019. Available at: https://www.researchgate.net/publication/334204965_Populism_and_Foreign_Aid. Accessed on 2.7.2020.

[CR26] Inglehart R, Norris P. Trump, brexit, and the rise of populism: economic have-nots and cultural backlash. HKS Faculty Research Working Paper Series RWP16–026026, August 2016. Available at: https://www.hks.harvard.edu/publications/trump-brexit-and-rise-populism-economic-have-nots-and-cultural-backlash. Accessed on 2.7.2020.

[CR27] ISSP. International Social Survey Programme: Citizenship II—ISSP 2014. GESIS Data Archive. 2016. https://search.gesis.org/research_data/ZA6670. Accessed 24.4.2020

[CR28] Jakupec V, Kelly M. Foreign Aid in the Age of Populism: Political Economy Analysis from Washington to Beijing. London: Routledge; 2019.

[CR29] Johnston M. Syndromes of Corruption: Wealth, Power, and Democracy. Cambridge: Cambridge University Press; 2005.

[CR30] Justesen MK, Bjørnskov C (2014). Exploiting the Poor: Bureaucratic Corruption and Poverty in Africa. World Dev.

[CR31] Karklins R. The System Made Me Do It: Corruption in Post-Communist Societies. Armonk, NY: M. E. Sharpe; 2005.

[CR32] Kopczuk W, Slemrod J, Yitzhaki S (2005). The limitations of decentralized world redistribution: An optimal taxation approach. Eur Econ Rev.

[CR33] Kotzian P (2014). Good Governance and Norms of Citizenship: An Investigation into the System- and Individual-Level Determinants of Attachment to Civic Norms. Am J Econ Sociol.

[CR34] Maeda K, Ziegfeld A (2015). Socioeconomic status and corruption perceptions around the world. RAP.

[CR35] Miller D, Banting K, Kymlicka W (2017). The Strains of Commitment: The Political Sources of Solidarity in Diverse Societies.

[CR36] Morgan J, Kelly NJ (2010). Explaining Public Attitudes toward Fighting Inequality in Latin America. PPP.

[CR37] Mudde C, Rovira Kaltwasser C (2012). Exclusionary vs. Inclusionary Populism: Comparing Contemporary Europe and Latin America. Gov Oppos.

[CR38] Noël A, Thérien J (2002). Public Opinion and Global Justice. Comp Polit Stud.

[CR39] Ostrom E (1998). A Behavioural Approach to the Rational Choice Theory of Collection Action Presidential Address, American Political Science Association. Am Polit Sci Rev.

[CR40] Paxton P, Knack S (2012). Individual and country-level factors affecting support for foreign aid. Int Polit Sci Rev.

[CR41] Putnam RD. Bowling Alone: The Collapse and Revival of American Community. New York: Simon & Schuster; 2000.

[CR42] Peyton K. Does Trust in Government Increase Support for Redistribution? Evidence from Randomized Survey Experiments. Am Polit Sci Rev. 2020;114(2):596–602. 10.1017/S0003055420000076.

[CR43] Putnam RD (2007). E Pluribus Unum: Diversity and Community in the Twenty-first Century The 2006 Johan Skytte Prize Lecture. Scand Polit Stud.

[CR44] QoG. The Quality of Government Standard Dataset, version Jan 20. 2020. https://www.gu.se/en/quality-government/qog-data/data-downloads/dataarchive. Accessed 12.5.2020.

[CR45] Rothstein B, Stolle D (2008). The State and Social Capital: An Institutional Theory of Generalized Trust. Comp Polit.

[CR46] Rothstein B, Uslaner E (2005). All for all - Equality, corruption, and social trust. World Polit.

[CR47] Rothstein B, Eek D (2009). Political Corruption and Social Trust: An Experimental Approach. Ration Soc.

[CR48] Rothstein B, Samanni M, Teorell J (2012). Explaining the welfare state: Power resources vs. the Quality of Government. Eur Polit Sci Rev.

[CR49] Rothstein B (2013). Corruption and Social Trust: Why the Fish Rots from the Head Down. Soc Res.

[CR50] Rothstein B. Solidarity, diversity and the quality of government. In: Banting K, Kymlicka W, editors. The Strains of Commitment: The Political Sources of Solidarity in Diverse Societies. Oxford: Oxford University Press; 2017. p. 300–26.

[CR51] Svallfors S (2013). Government quality, egalitarianism, and attitudes to taxes and social spending: A European comparison. Eur Polit Sci Rev.

[CR52] Tourangeau R, Yan T (2007). Sensitive Questions in Surveys. Psychol Bull.

[CR53] Tranow U. Solidarity as a System of Norms. In: Althammer J, Neumärker B, Nothelle-Wildfeuer U, editors. Solidarity in Open Societies. Wiesbaden: Springer VS; 2019. p. 25–55.

[CR54] UN News. ‘Global solidarity’ needed, to find affordable, accessible COVID-19 vaccine. June 4, 2020. https://news.un.org/en/story/2020/06/1065622. Accessed 4.8.2020.

[CR55] UNU-WIDER. World Income Inequality Database (WIID3.4). 2017. https://www.wider.unu.edu/database/world-income-inequality-database-wiid34. Accessed 14.5.2020.

[CR56] Uslaner EM (2008). Corruption.

[CR57] Van de Walle SGJ, Huberts LWJC, Maesschalk J, Jurkiewicz CL (2008). Perceptions of Corruption as Distrust? Cause and Effect in Attitudes towards Government. Ethics And Integrity And The Politics Of Governance.

[CR58] VanHeuvelen T (2017). Unequal views of inequality: Cross-national support for redistribution 1985–2011. Soc Sci Res.

[CR59] V-Dem. Country-Year/Country-Date Dataset v8. 2018. https://www.v-dem.net/dsarchive.html. Accessed 18.5.2020.

[CR60] Dahlström C, Lindvall J, Rothstein B (2013). Corruption, Bureaucratic Failure and Social Policy Priorities. Polit Stud-London.

[CR61] Verbeek B, Zaslove A, Rovira Kaltwasser C, Taggart P, Ochoa Espejo P, Ostiguy P (2017). Populism and Foreign Policy. The Oxford Handbook of Populism.

[CR62] Warren ME (2006). Political Corruption as Duplicitous Exclusion. PS Polit Sci Polit.

[CR63] Zaslove A, Geurkink B, Jacobs K, Akkerman A. Power to the people? Populism, democracy, and political participation: a citizen's perspective. West Eur Polit 2020;ahead-of-print(ahead-of-print). 2020:1–25. 10.1080/01402382.2020.1776490

[CR64] Zmerli S (2010). Social Capital and Norms of Citizenship: An Ambiguous Relationship?. Am Behav Sci.

